# Supplementary material for: COMT Val/Met and Psychopathic Traits in Children and Adolescents: A Systematic Review and New Evidence of a Developmental Trajectory toward Psychopathy
Source: Int J Mol Sci. 2022 Feb 4;23(3):1782. doi: 10.3390/ijms23031782 (PMC8836546; doi:10.3390/ijms23031782)
Supplement: Supplementary file 1 [file ijms-23-01782-s001.zip › Supplementary Material S1.pdf]

Supplementary Material S1

Results for the Kruskal-Wallis tests for Val/Val vs Val/Met vs Met/Met:

Male >= 13:
- CBCL Aggressive Behavior: $p = 0.021$
- CBCL Oppositional Defiant Problems: $p = 0.037$
- PSD Callous-Unemotional Traits subscale: $p = 0.022$

Male < 13:
- CBCL Oppositional Defiant Problems: $p = 0.048$
- CBCL Conduct Problems: $p = 0.021$
- PSD Impulsivity subscale: $p = 0.040$

Female >= 13:
- N/A

Female < 13:
- N/A

Dunn Post Hoc tests:

0 -> Val/Val
1 -> Val/Met
2 -> Met/Met

Male >= 13:
- CBCL Aggressive Behavior: $p = 0.021$
    - 0 vs 1: $p = 0.0379$
    - 0 vs 2: $p = 0.1575$
    - 1 vs 2: $p = 0.0044$
- CBCL Oppositional Defiant Problems: $p = 0.037$
    - 0 vs 1: $p = 0.0220$
    - 0 vs 2: $p = 0.4939$
    - 1 vs 2: $p = 0.0175$
- PSD Callous-Unemotional Traits subscale: $p = 0.022$
    - 0 vs 1: $p = 0.3111$
    - 0 vs 2: $p = 0.0166$
    - 1 vs 2: $p = 0.0166$

Male < 13:
- CBCL Oppositional Defiant Problems: $p = 0.048$
    - 0 vs 1: $p = 0.0146$

    - 0 vs 2: $p = 0.2721$
    - 1 vs 2: $p = 0.0362$
- CBCL Conduct Problems: $p = 0.021$
    - 0 vs 1: $p = 0.0399$
    - 0 vs 2: $p = 0.3279$
    - 1 vs 2: $p = 0.0044$
- PSD Impulsivity subscale: $p = 0.040$
    - 0 vs 1: $p = 0.4366$
    - 0 vs 2: $p = 0.0258$
    - 1 vs 2: $p = 0.0085$

Female >= 13:
- N/A

Female < 13:
- N/A
