# Supplementary material for: COMT Val/Met and Psychopathic Traits in Children and Adolescents: A Systematic Review and New Evidence of a Developmental Trajectory toward Psychopathy
Source: Int J Mol Sci. 2022 Feb 4;23(3):1782. doi: 10.3390/ijms23031782 (PMC8836546; doi:10.3390/ijms23031782)
Supplement: Supplementary file 1 [file ijms-23-01782-s001.zip › Supplementary Table S1.pdf]

Supplementary Table S1. Sample Characteristics for Males and Females Below and Above Age 13, based on *COMT* Val158Met Val and Met alleles

	Males						Females					
	Below age 13 (*n* = 81)			Above age 13 (*n* =59)			Below age 13 (*n* = 70)			Above age 13 (*n* = 70)		
	Val/Val (*n* = 22)	Val/Met (*n* = 36)	Met/Met (*n* = 23)	Val/Val (*n* = 17)	Val/Met (*n* = 30)	Met/Met (*n* = 12)	Val/Va l (*n* = 22)	Val/Met (*n* = 30)	Met/Met (*n* = 18)	Val/Val (*n* = 24)	Val/Met (*n* = 34)	Val/Val (*n* = 22)
Mean age (S.D)	9.95 (2.07)	10.43 (1.88)	10.03 (2.28)	14.59 (1.35)	15.03 (1.70)	14.25 (1.05)	10.70 (1.85)	10.42 (1.83)	9.84 (2.12)	14.59 (1.24)	14.95 (1.28)	15.07 (1.45)
Mean CBCL Aggressive Behavior (S.D)	66.3 (16.59)	65.31 (16.15)	72.52 (16.18)	**66.76 (14.29)**	**75 (12.76)**	**61.91 (12.32)**	56.09 (8.65)	64.38 (14.12)	61.63 (15.77)	70.14 (14.60)	71.83 (15.87)	66.45 (15.04)
Mean CBCL Attention Deficit / Hyperactivity (S.D)	63.25 (12.86)	57.4 (10.93)	63.77 (8.70)	61.5 (8.80)	65.38 (7.17)	58.43 (9.88)	55.8 (5.68)	61.33 (10.55)	59.5 (12.45)	66.4 (10.59)	68.57 (9.63)	63.31 (22.53)
Mean CBCL Oppositional Defiant Problems (S.D)	**68.75 (12.16)**	**58.35 (11.55)**	**65.92 (11.62)**	**60.33 (12.40)**	**70.56 (7.85)**	**61.14 (8.71)**	55.4 (6.59)	61.39 (10.48)	61.2 (13.35)	65.4 (10.47)	69.43 (10.76)	66.23 (12.19)
Mean CBCL Conduct Problems (S.D)	**68.5 (13.83)**	**58.65 (13.00)**	**71.08 (13.80)**	63.17 (15.21)	72.69 (9.88)	62.29 (10.97)	53.13 (5.78)	62 (12.69)	59.1 (12.44)	73.2 (16)	75.14 (12.03)	69.69 (13.78)
Mean PSD Narcissism subscale (S.D)	0.56 (0.45)	0.61 (0.53)	0.81 (0.63)	0.80 (0.50)	1.05 (0.52)	0.64 (0.45)	0.41 (0.31)	0.52 (0.45)	0.31 (0.31)	0.95 (0.68)	0.84 (0.64)	0.63 (0.60)
Mean PSD Impulsivity subscale (S.D)	**0.86 (0.62)**	**0.88 (0.67)**	**1.25 (0.48)**	1.22 (0.63)	1.35 (0.56)	0.82 (0.67)	0.49 (0.34)	0.82 (0.65)	0.77 (0.55)	1.2 (0.70)	1.24 (0.70)	1.03 (0.68)

Mean PSD Callous-Unemotional Traits subscale (S.D)	0.9 (0.36)	0.88 (0.46)	0.89 (0.52)	**1.05 (0.46)**	**1.12 (0.29)**	**0.78 (0.24)**	0.76 (0.37)	0.81 (0.38)	0.93 (0.43)	0.9 (0.55)	0.95 (0.45)	0.86 (0.43)

note: **bold:** *p* <0.05
